# Design and Experimental Evaluation of Modified Square Loop Feeding for UHF RFID Tags

**DOI:** 10.1371/journal.pone.0132530

**Published:** 2015-07-15

**Authors:** Md. Rokunuzzaman, Mohammad Tariqul Islam, Haslina Arshad, Mandeep Jit Singh, Norbahiah Misran

**Affiliations:** 1 Department of Electrical Electronic and Systems Engineering, Faculty of Engineering and Built Environment, Universiti Kebangsaan Malaysia (UKM), Bangi, Selangor, Malaysia; 2 School of Information Technology, Faculty of Information Science and Technology, Universiti Kebangsaan Malaysia (UKM), Bangi, Selangor, Malaysia; Aristotle University of Thessaloniki, GREECE

## Abstract

This paper addresses the performance evaluation of a modified square loop antenna design for UHF RFID applications that is excited through a narrow feed line connected to a square loop, an impedance matching network. The square loop dimensions are modified to reach a conjugate impedance matching. A gap is fixed between the feed-lines to link the chip. To achieve impedance matching, the structures of the feed-line are optimized accordingly. In addition, the antenna consists of a straightforward geometry. An 11.9-meter maximum read range is achieved using a compact size of 80 × 44 mm^2^ and 3.2 W for the effective isotropic radiated power. Additional findings reveal that the proposed tag antenna is able to provide a stable resonance response in the near field of a large metallic surface.

## Introduction

Radio Frequency Identification or RFID technology is currently receiving considerable attention due to the increasing technological support of various RFID applications. This is particularly true of the ultra-high frequency (UHF) RFID system that has now reached far beyond the management and tracking of the supply chain [1]. RFID consists of a basic structure that integrates the application-specific integrated circuit (ASIC), a reader, a tag antenna, a writer to write the codes encoded in the ASIC, and a computer for process monitoring. The RFID tag antenna’s ASIC has a complicated impedance. In a tiny development environment, it is hard for IC designers to work with a 50-Ω impedance-matched IC design. All of the ICs contain capacitances that offer complicated impedances, which in turn complicate the RFID tag antenna design and measurements procedure to start with. The tag antenna impedance needs to be conjugate matched with the ASIC chip to achieve antenna output.

Two 2D and a 3D monopole tag antenna for UHF RFID are demonstrated in [2]. The antenna possesses a 5-meter average reading range. Nevertheless, the antenna is designed using the costly Rogers RO4350B substrate and does not include the UHF RFID frequency spectrum entity. Two U-shaped slots are implemented using an asymmetric tag antenna in [3] to achieve the RFID band. The antenna’s read range is at its maximum, which is 4.3 meters, when a metal plate is introduced within the antenna’s near field. The antenna, however, has a narrow 910 MHz bandwidth. The technique of wideband impedance matching using the inductively coupled feed structure is demonstrated in [4]. The dimensions are 200 × 160 mm^2^. However, the antenna has a narrow reflection coefficient of -10 dB at the UHF RFID band. A planar structure of a UHF RFID tag antenna using an open stub feed is demonstrated in [5]. The antenna has a 6-meter read range. Moreover, the length and thickness are 106.5 mm and 3 mm, respectively, which is bulky for RFID applications. The resonance response of the antenna encompasses just a part of the UHF RFID band. Polyethylene terephthalate (PET) is used to create a structure that is flexible and thin for the RFID tag antenna in [6]. A narrow resonance response is discovered in the antenna’s simulation, which has a tendency to shift to 900 MHz in the measured outcome. An antenna based on the planar dipole is demonstrated in [7]. The antenna is shaped like a bowtie, with an inductive coil mounted at the mid-range of the bowtie to conjugate match the antenna’s impedance with the IC ship. Nevertheless, the antenna only partially covers the UHF RFID band. An antenna with a passive UHF tag for near-body applications is demonstrated in [8]. A model of a human torso is introduced in the Computer Simulation Technology (CST) environment, and the antenna is applied over the model to simulate the effect. The antenna’s read range varies depending on the material on which it is mounted. The antenna’s ground plane size is 137 × 32 mm^2^ with a 5-meter read range. A passive RFID tag antenna design that is bowtie-shaped is demonstrated in [9]. An artificial magnetic conductor (AMC) is used at the antenna’s ground plane so that it is applicable for metallic surfaces. Here, the antenna geometry’s dimensions are 140 × 80 mm^2^ with a 50-mm thickness. A normal mode helical antenna is revealed with a gain of -0.5 dBd in [10]. Nonetheless, the antenna’s structure is able to change during use in RFID applications, hence transforming the characteristics of the antenna. A cross-sectional slot is etched out from the mid portion of the rectangular microstrip line to gain a circular polarization, as shown in [11]. A shorting pin is used to short the RFID chip. However, the shorting pin is a complicated structure under consideration in the design. A tag antenna with a broadband characteristic is revealed in [12], contemplating a -3 dB scale. However, the antenna’s performance dramatically degrades at the near field’s metallic surface.

This study proposes a passive microstrip tag antenna with a vertically omnidirectional radiation pattern. The antenna encompasses the universal UHF RFID band (860 MHz-960 MHz). It exhibits a low-profile, compact, and robust structure that makes it appropriate for RFID applications. The antenna’s substrate material is used widely in low-cost FR4 (Fibre-Reinforced plastic) with a permittivity of Ɛ_FR4_ = 4.6, thickness of 1.6 mm, and dielectric loss tangent of 0.02. The antenna’s square loop and feed-line are optimized to reach conjugate impedance matching with the ASIC chip’s impedance. The ASIC chip that was chosen for the proposed tag antenna is the NXP SL3S1213 UCODE G2iL UHF RFID chip from the NXP (Next Experience) [13]. An antenna prototype with a size of 0.224 λ is fabricated to validate the antenna’s simulation. The antenna’s design will be discussed in the following section. Section 3 will explain the parametric studies. Section 4 will be used to introduce the fabricated antenna, and section 5 will expound on the proposed antenna’s performance. The conclusion is derived in section 6.

## Antenna Design

**Fig 1:**
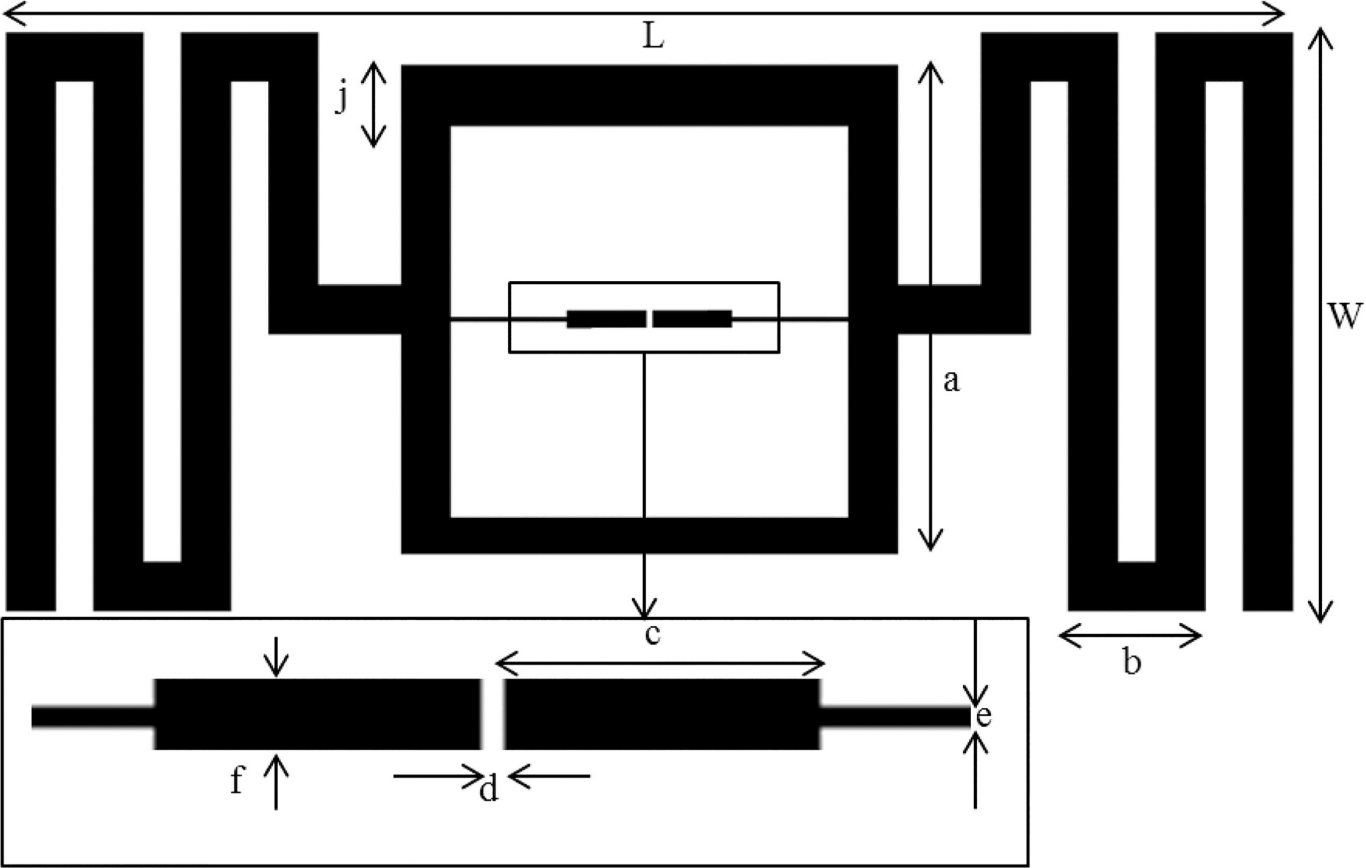
Dimensions of the proposed tag antenna where, *L* = 80, *W* = 44, *h* = 1.6, *a* = 30, *b* = 8.3, *c* = 10, *d* = 0.335, *e* = 0.3, *f* = 1.2, *j* = 3 (in mm).

Fig 1 demonstrates the antenna’s basic structure. The proposed tag antenna possesses a larger substrate at the outer boundary than the antenna’s basic structure. This structure is reached so that the outer boundary cannot damage the antenna’s interior dimensions. It allows the proposed RFID tag antenna design to be robust and to have a less harmful surveying portability. The FR4 substrate with the thickness of h = 1.6 mm is selected for the antenna to achieve compactness and durability. The microstrip feed-lines are positioned near each other (0.335-mm gap in-between) to mount an NXP SL3S1213 UCODE G2iL UHF RFID chip that touches the edge of the feed-lines. The antenna’s outer structure is meandered to reach the bandwidth performance within the size limitation. The meandered lines possess a unique distance from each other, which allows the design to be easily realized. In comparison with a single-loop meandered line, a double loop configuration increases the antenna’s bandwidth [14]. A square loop that matches the conjugate impedance of the antenna with tag chip impedance is introduced. A narrow microstrip line with a 0.3-mm width connects the IC chip to the impedance matching loop. The meandered lines are connected at the mid portion of the square loop to contain the dipole-like radiation responses. The length, L, and width, W, of the substrate are 80 mm and 44 mm, respectively. The UHF RFID chip’s impedance is 23-j224 Ω at 915 MHz. The ASIC’s impedance slowly changes with the frequency change within the universal UHF band. A deeper analysis of the impedance measurements of the UHF RFID chip can be observed in [15]. The power transmission coefficient, τ, can be calculated using the equation as shown below:

$$\tau =\frac{4R_{IC}R_{antenna}}{{\left|Z_{IC}+Z_{antenna}\right|}^{2}}$$ (1)

where R_IC_ = the real impedance of the IC chip and R_antenna_ = the real impedance of the proposed tag antenna. Z_IC_ and Z_antenna_ are the complex impedances of the IC and antenna, respectively. It can be observed from the equation that a proper conjugate matching of the impedance will provide the antenna’s highest power transmission.

## Parametric Studies

The antenna’s parameters are analyzed to characterize the antenna’s impedance response for various parametric values and to optimize the antenna for the UHF RFID frequency. Because the meandered lines assist in the propagation of the wave, the meandered line’s dimensions are kept constant throughout the analysis of the parameters. The feed-line structure has an essential function in matching the UHF RFID tag antenna with the ASIC’s conjugate impedance. One of the key objectives in designing the passive tag antenna is cost-effectiveness. Although a high dielectric constant substrate is used, the antenna’s dimension can be minimized. Nevertheless, the most essential characteristic for the passive tag antenna is cost-effectiveness. The proposed antenna is designed using the FR4 substrate, which is durable and has constant performance in most environments. The FR4 is also cost effective in comparison to the RT/duroid substrates.

**Fig 2:**
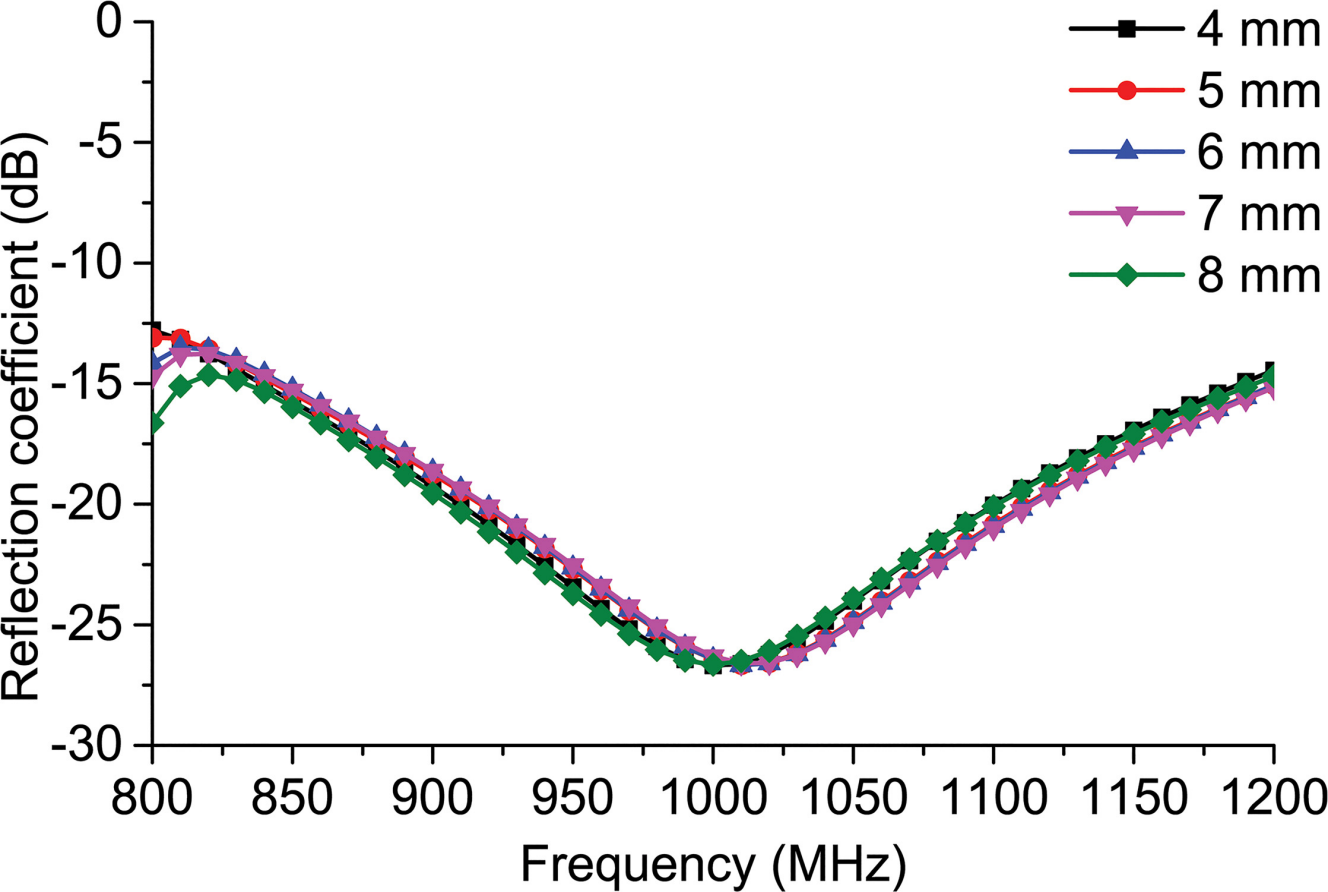
Reflection coefficient response to the parametric change in square loop thickness ‘*j*’.

**Fig 3:**
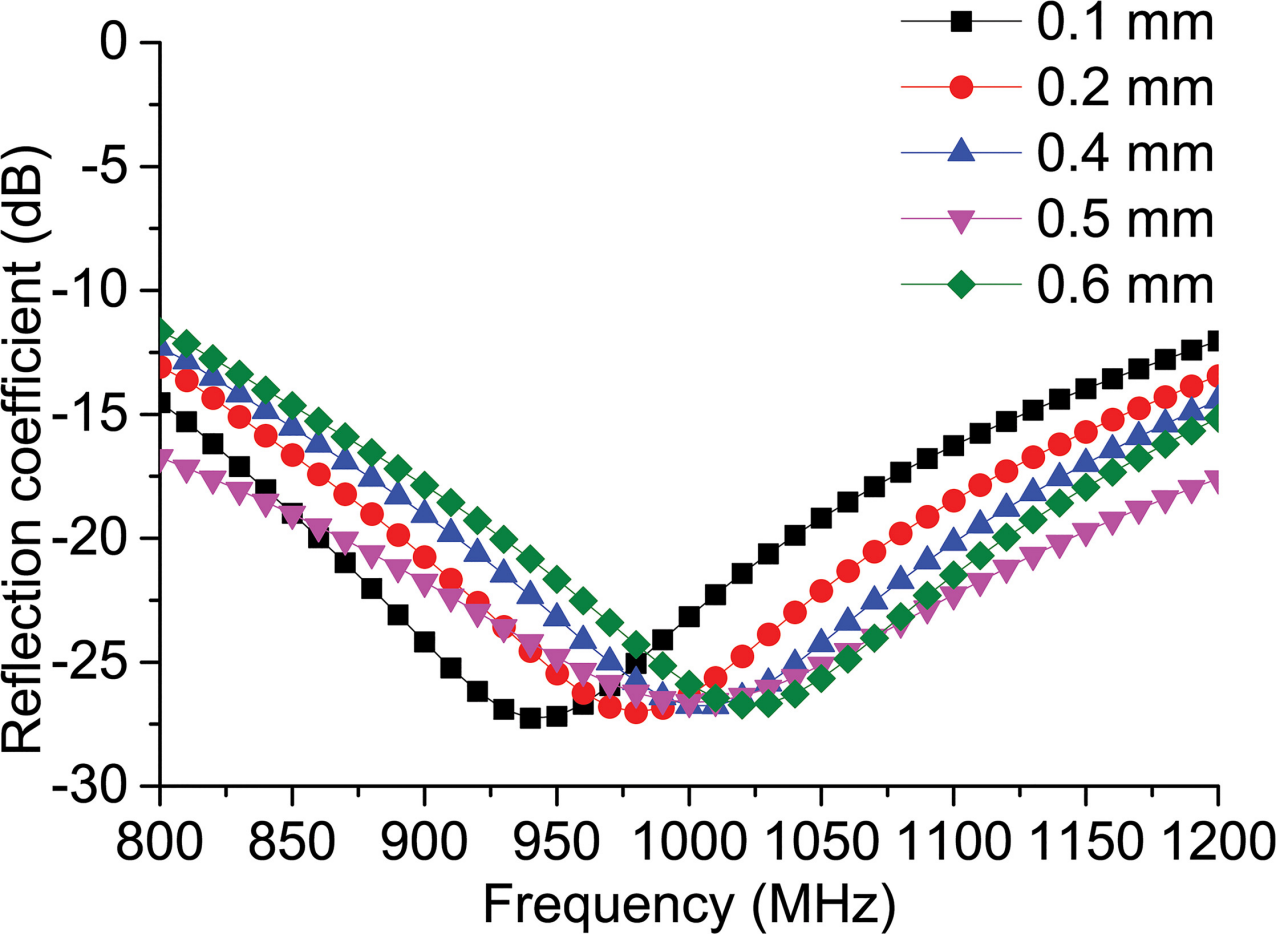
Reflection coefficient response to the parametric change in feed-line connector width ‘*e*’.

Fig 2 demonstrates the antenna’s parametric analysis along with the change in the square loop’s overall thickness ‘*j*.’ The figure reveals that after a change in thickness of over 3 mm, not much change is observed in the reflection coefficient. The feed-line connector connects the chip feed-line to the square loop. In addition to connecting the feed-line to the square loop, it also contributes to the matching of the conjugate impedance of the antenna with the ASIC. Fig 3 reveals the change in the reflection coefficient when the feed-line connector width ‘*e*’ is changed. It can be observed from the figure that the change in width changes the reflection coefficient. The antenna’s resonance has a tendency to shift towards a frequency of 925 MHz for *e* = 0.1 mm. The resonance moves at a frequency of 975 MHz at *e* = 0.2 mm. The antenna’s resonance is inclined to move outwards from the UHF RFID band when *e* = 0.4 mm. The resonance moves in the direction of the frequency increment at *e* = 0.5 mm. The resonance response is displaced more towards a higher frequency in comparison to the UHF RFID when *e* = 0.6 mm. It can be observed from this parametric study that the antenna is tunable for higher frequencies, offering unlimited design flexibility if needed.

**Fig 4:**
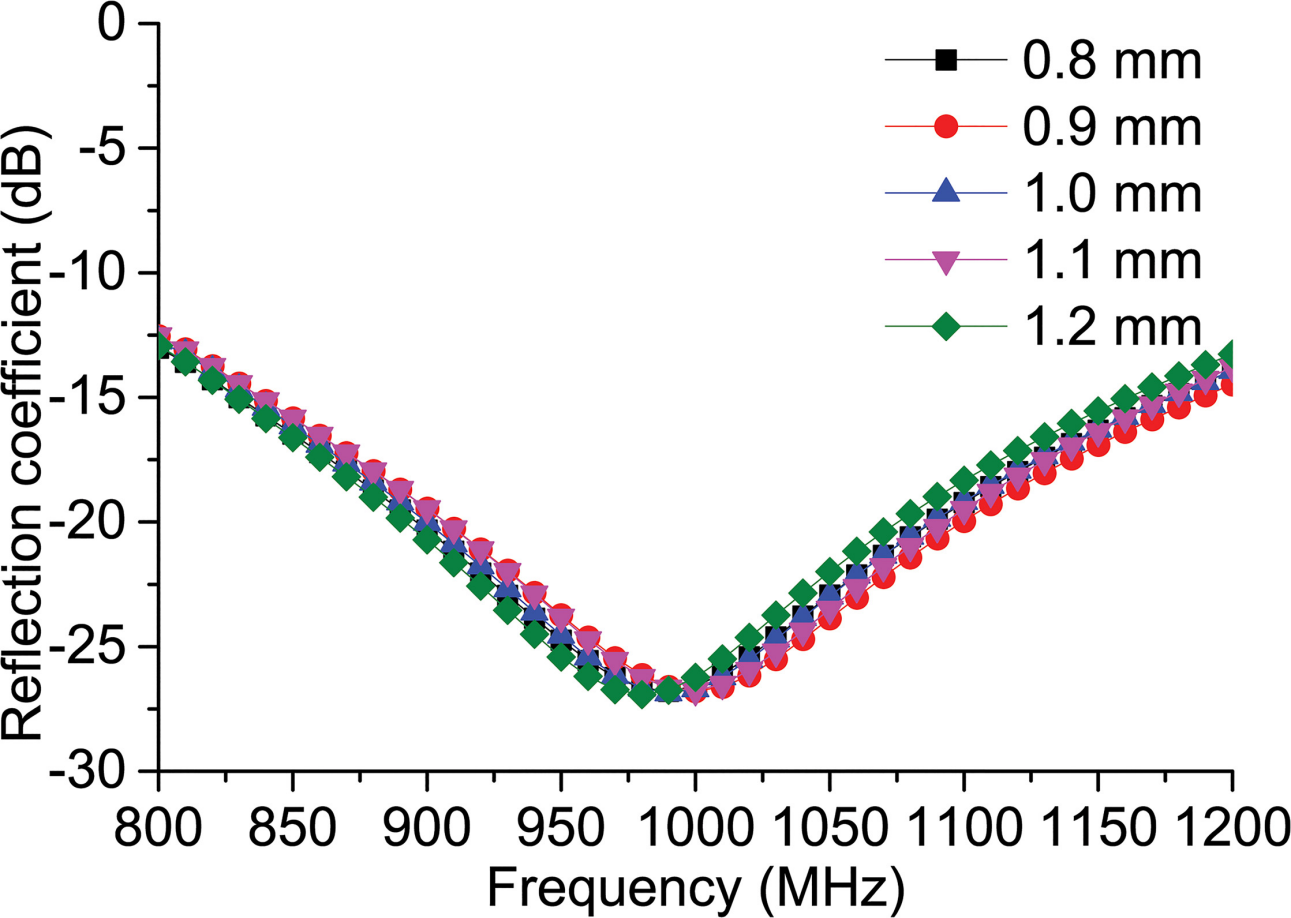
Reflection coefficient response to the parametric change in feed-line width ‘*f*’.

The antenna’s feed-line is a critical design parameter because the antenna’s impedance matching is highly dependent on the structure of the feed-line. In this tag antenna design, however, the impedance is matched using the feed-line, the connector, and the square loop impedance matching, which allows the antenna’s performance to be less affected by just one parameter. Fig 4 demonstrates the antenna’s resonance response when the width ‘*f*’ of the feed-line is changed to different values while the other parameters are kept constant. Following the change of value of ‘*f*’ from 0.8 mm to 1.2 mm, it can be seen that the antenna’s resonance response remains almost constant throughout the UHF RFID frequency, affirming the fact that the antenna provides a stable performance within the UHF RFID frequency given a sudden change in the dimension.

**Fig 5:**
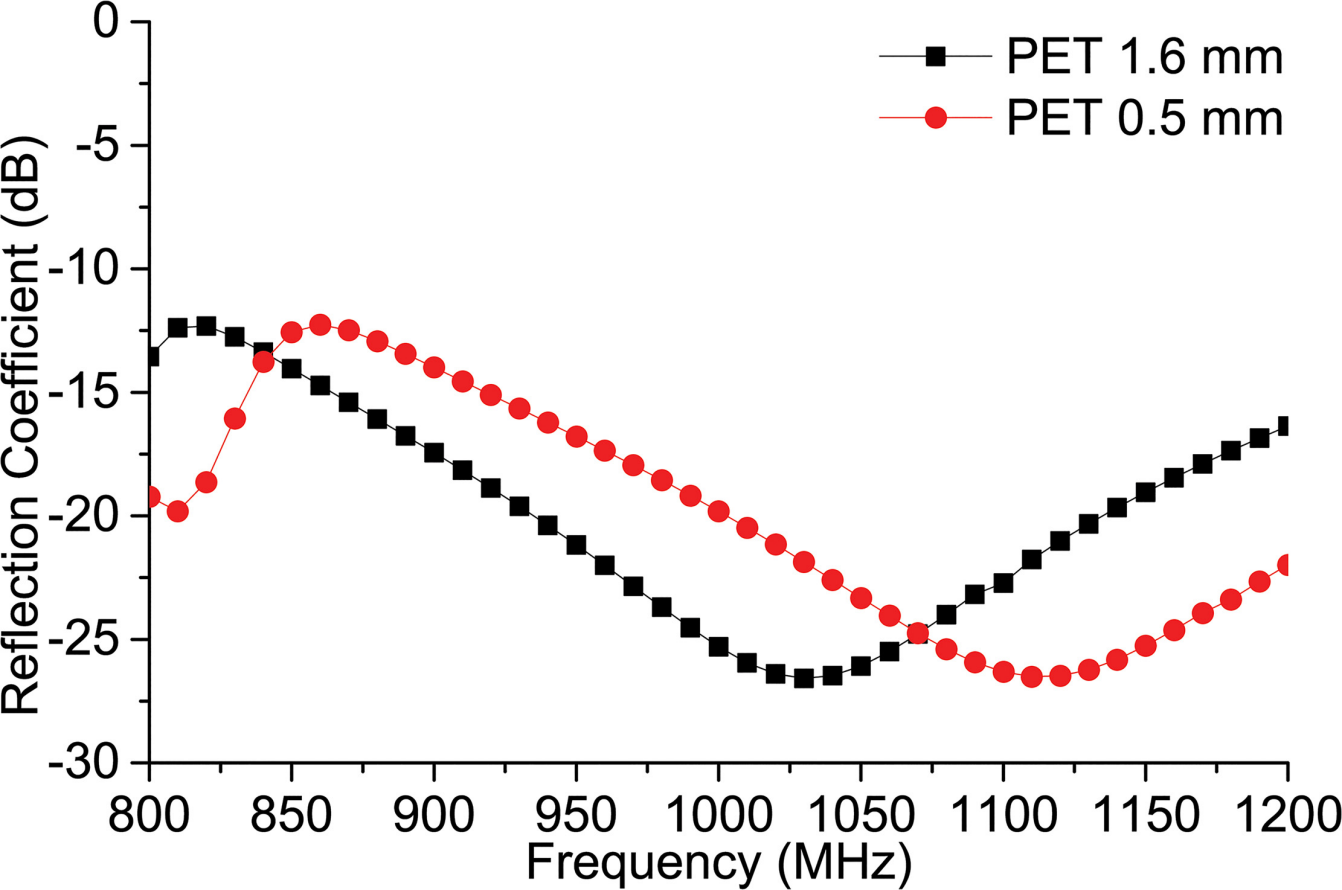
Reflection coefficient response for the flexible PET substrate of different thickness.

Fig 5 depicts the antenna resonance response when a flexible PET substrate (Ԑ_r_ = 3.4) is used instead of FR4. For two different thicknesses (1.6 mm and 0.5 mm), the resonance tends to shift slightly upwards. Nonetheless, the global UHF RFID band falls within the resonance band.

## Antenna Structure

**Fig 6:**
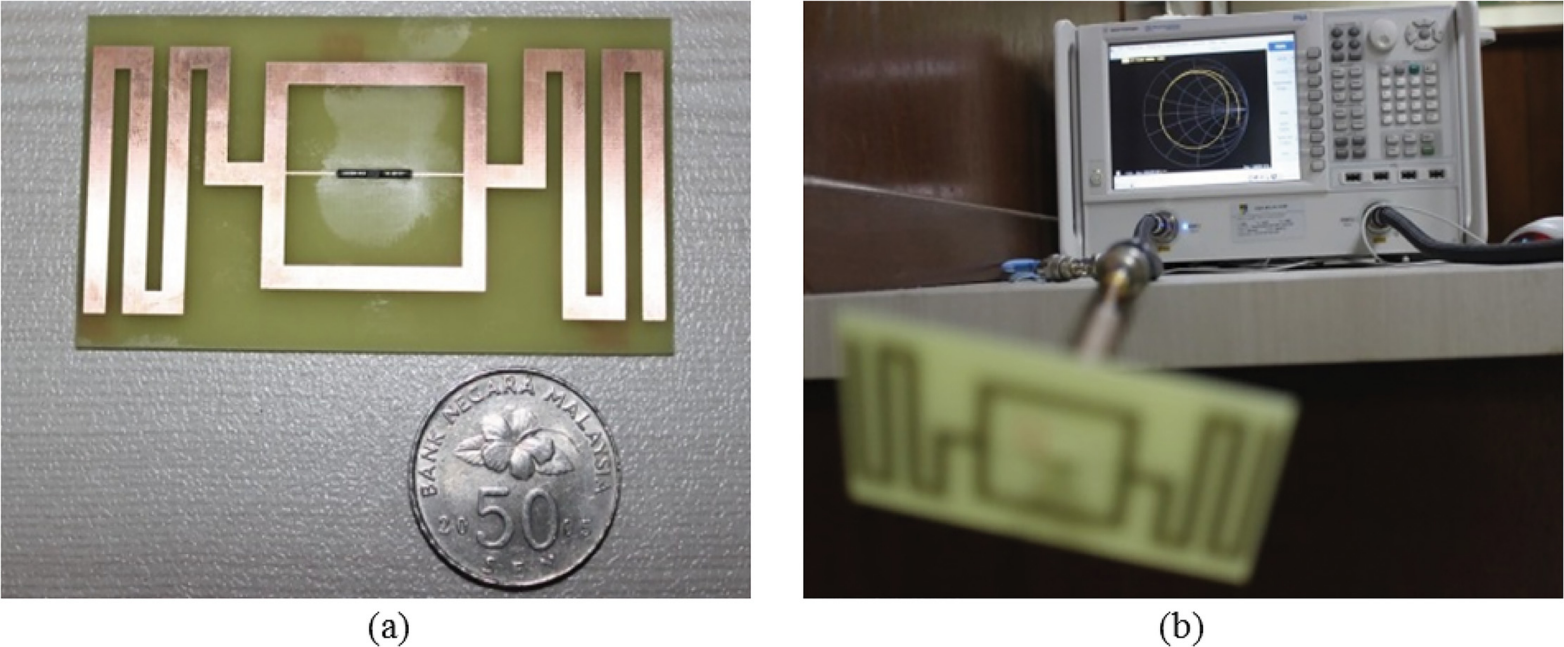
Fabricated antenna prototype and its measurement using balun.

**Fig 7:**
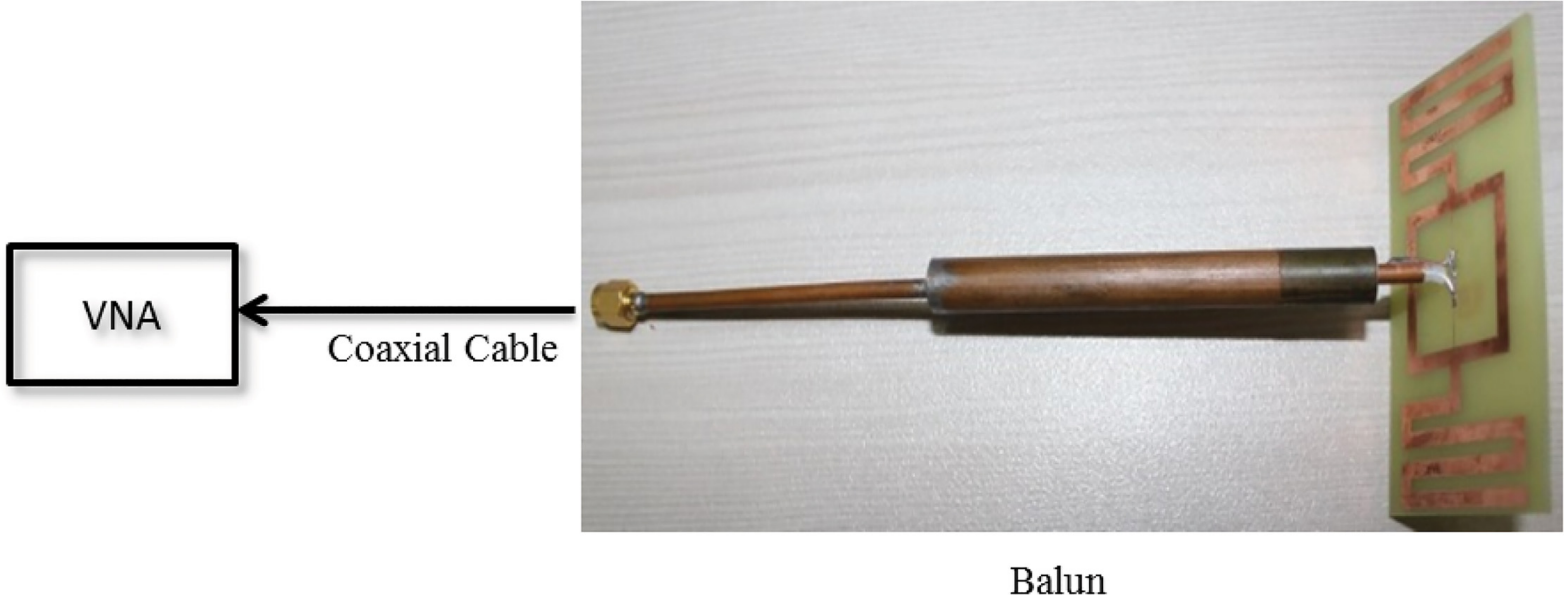
Impedance measurement setup.

Fig 6 reveals the proposed antenna with a size of 80 × 44 mm^2^. The structure is fabricated using the LPKF (S63) PCB prototyping machine on a low-cost FR4 substrate (loss tangent = 0.02) with 1.6-mm thickness and 4.6 relative permittivity. The substrate’s ground plane is etched out. The meandered microstrip lines are printed on both sides of the square loop with identical lengths, *W* = 44 mm. Two microstrip feed-lines of equal length, *c* = 10 mm, and width, *e* = 0.3 mm, are used to connect the tag chip and the square loop. The fabricated antenna is measured using the Agilent Technology vector network analyzer (VNA) with model no. N5227A. The antenna’s feed-line is connected to the VNA as depicted in Fig 6. The proposed antenna is symmetrical in nature. To measure the antenna using 50-Ω VNA, a balun of length *λ/4* is used as the connector between the proposed antenna and the VNA. The setup process for the impedance measurement is shown in Fig 7. One of the feed-lines of the antenna is connected with the coaxial cable inside the balun, and the other feed-line is grounded using the ground of the balun. Once the balun is soldered properly with the antenna feed-lines, the other ends of the balun are connected to the VNA and used to measure the 50-Ω impedance responses.

Generally, the reference impedance (*Z*_*0*_) for measurement of the reflection coefficient response is 50 Ω; however, for the tag antenna, the connector is different, and the reference impedance depends entirely on the conjugate impedance of the tag chip. To calculate the reflection coefficient of the antenna for the different reference impedance, the following equation is used [16]:

$$S_{11}=\frac{Z_{0}-Z_{a}^{*}}{Z_{0}+Z_{a}}$$ (2)

Here, *Z* ^*^_a_ is the conjugate of the antenna impedance *Z*_*a*_.

## Results and Discussion

**Fig 8:**
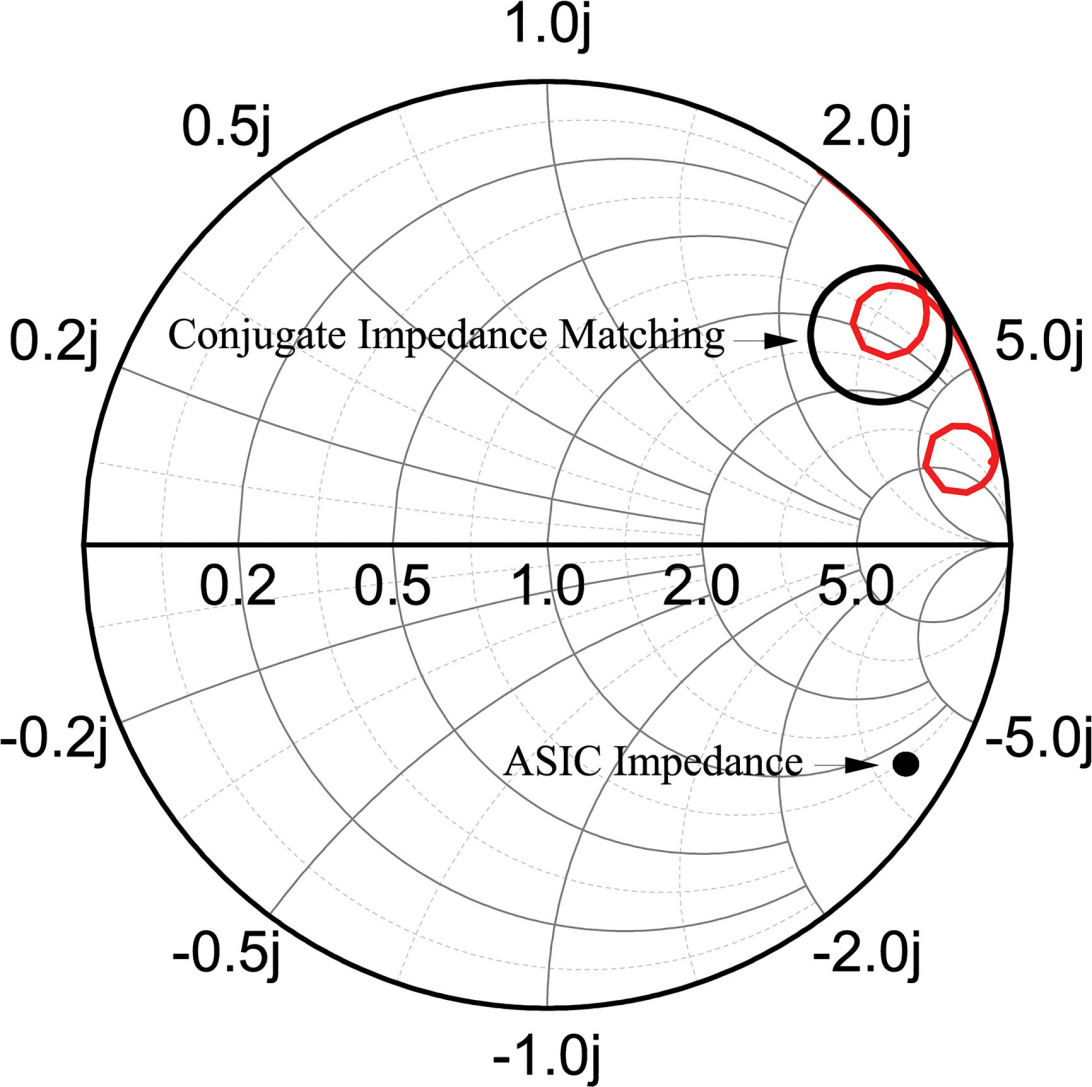
Conjugate impedance matching between ASIC and antenna.

**Fig 9:**
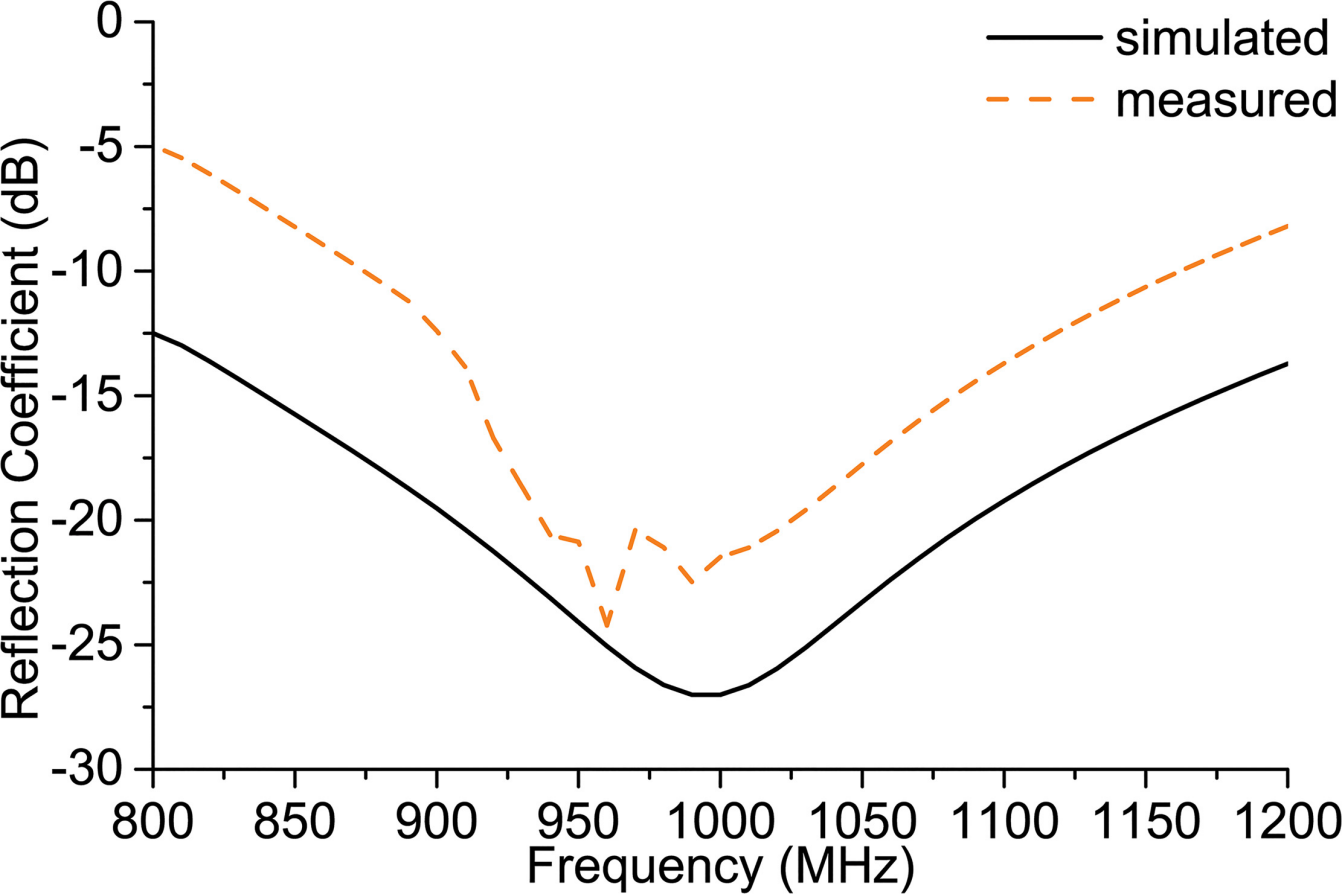
Reflection coefficient of the proposed antenna.

Fig 8 depicts the smith chart response of the antenna feed line. It can be observed that the loop created near the impedance 23+j224 Ω falls within the VSWR<2 circle, which confirms a broadband matching with the desired polarization. For the IC chip impedance of 23-j224 Ω, a conjugate impedance of 23+j224 Ω is achieved as the antenna’s input impedance to reach the response as demonstrated in Fig 9. The result shown in Fig 9 is normalized in terms of the ASIC impedance. The figure demonstrates that the simulated reflection coefficient is lower than -10 dB at the universal UHF RFID operating frequency, showing that the reflected power is lower than 10% at the operating frequency. Although the simulated result reveals a bandwidth performance that is wider than the UHF RFID band, the measured reflection coefficient has a wideband performance that is narrow in comparison to the measured data retrieved from the simulation software. The calculated antenna’s -10 dB bandwidth ranges from 870 MHz to 1160 MHz with a maximum resonance of -24 dB at 960 MHz.

**Fig 10:**
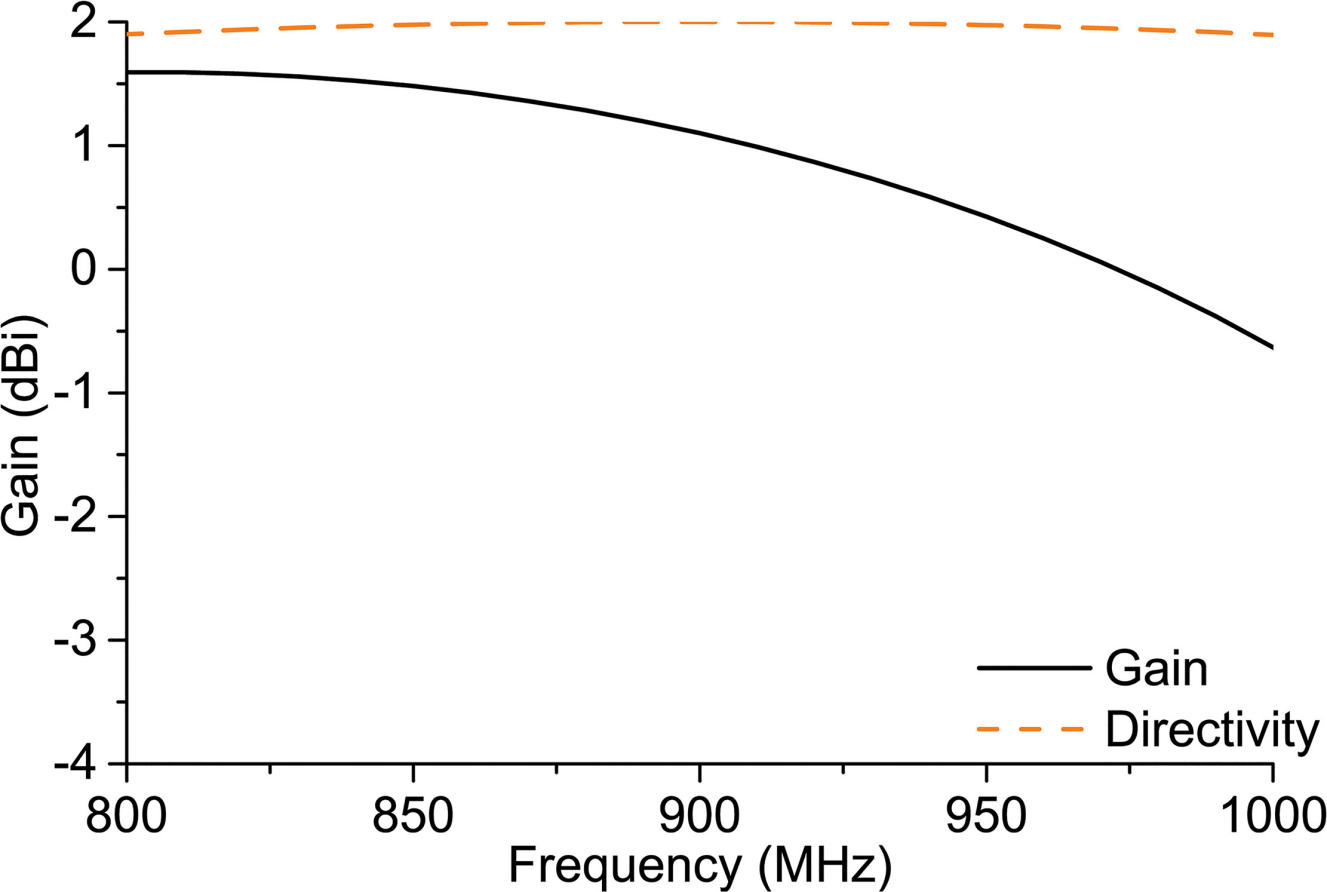
Directivity and realized gain.

Fig 10 reveals the antenna’s realized gain and directivity. The directivity pattern depicts that the directivity is almost constant throughout the UHF RFID band. An average 1.8-dBi directivity is reached within the UHF RFID band. Nevertheless, the antenna’s realized gain has the tendency to gradually reduce with the increase in frequency within the UHF RFID band. The highest gain of 1.43 dBi is achieved at 860 MHz. The lowest gain is found at the 960-MHz frequency of the UHF RFID band with a 0.25-dBi value. Although the gain is decaying with the increment of frequency, the directivity tends to be stable. The gain within the frequency range 840 MHz—900 MHz is stable above 1 dBi. The read range of the proposed antenna depicts the gain performance in a practical environment. Additionally, the direction of the antenna radiation confirms that there is negligible loss at the side-lobes.

**Fig 11:**
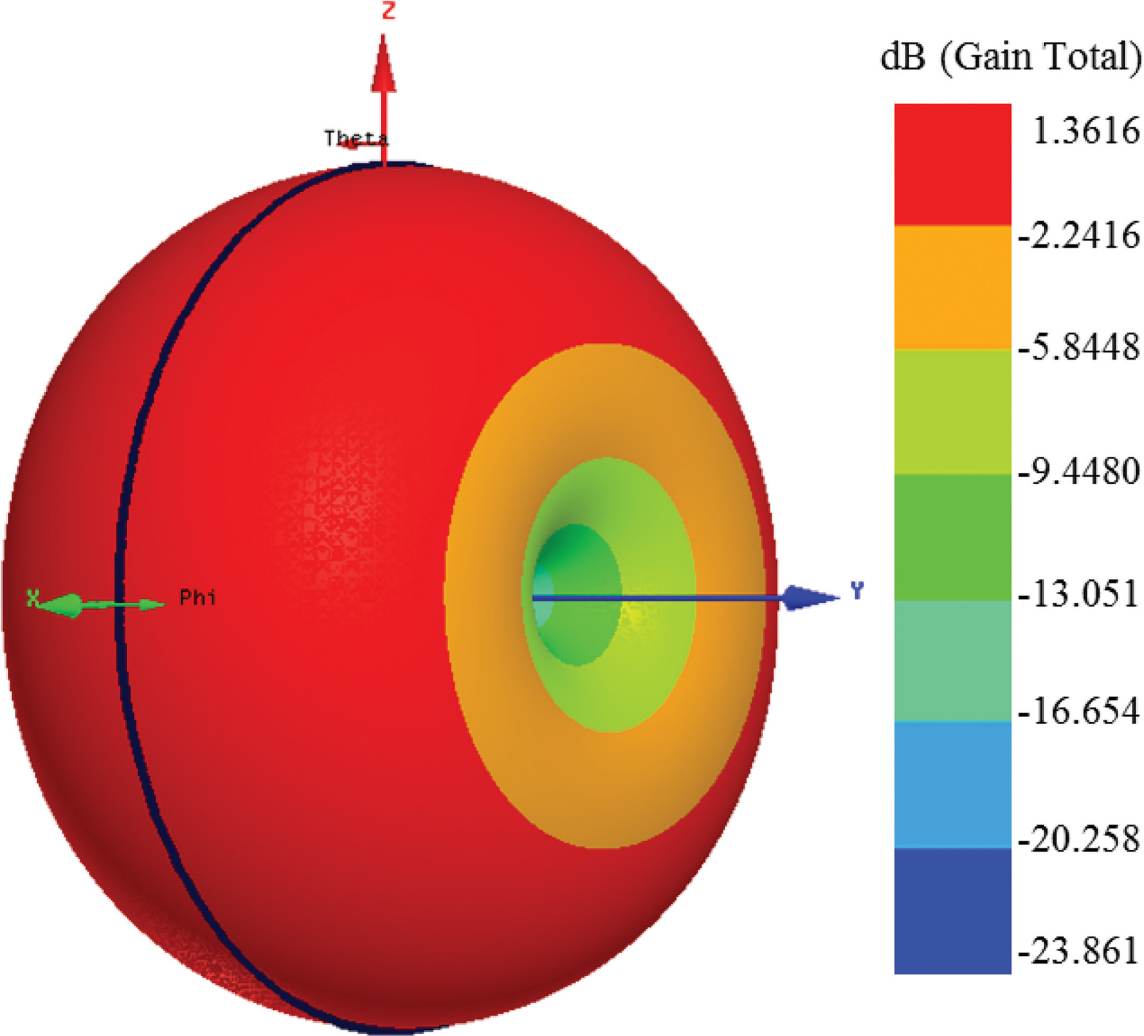
Simulated 3D far field radiation pattern of the antenna at 915 MHz.

Fig 11 demonstrates the antenna’s far field radiation pattern at a frequency of 915 MHz. The 3D radiation pattern is shaped like a donut in compliance with the dipole-type radiation pattern of the proposed antenna. The figure depicts that the radiation beam is concentrated towards the normal antenna dimension that is demonstrated in red. The peak 3D radiation pattern is 1.3616 dB, which includes most of the donut-shaped pattern. Two nulls are presented at both sides of the Y-axis.

**Fig 12:**
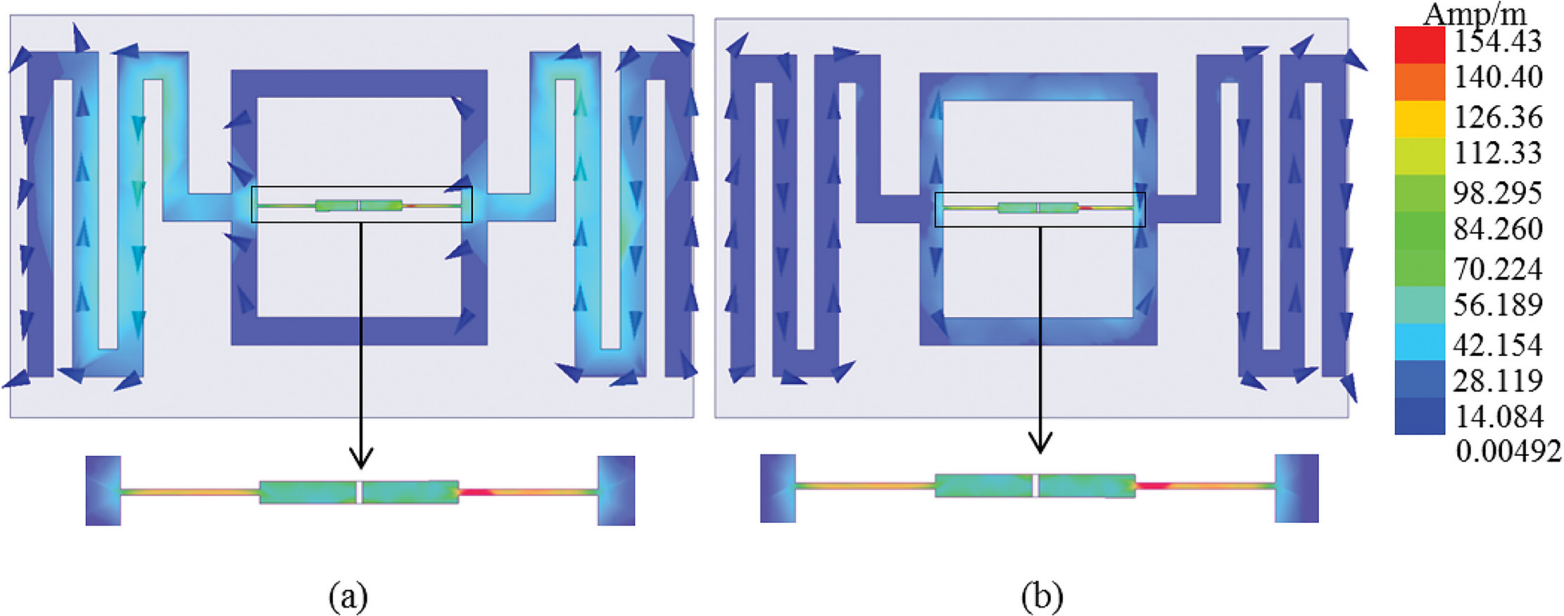
Current distribution of the antenna at (a) 0° and (b) 90° of 915 MHz frequency.

Fig 12 demonstrates the antenna’s present pattern of distribution at 915 MHz (0° and 90°). Fig 12 (A) reveals that the highest value of the present distribution can be seen at the surface of the narrow microstrip line (feed-line) IC chip connector. The current flows from the positive Y-axis direction towards the direction of the negative Y-axis. At the antenna’s dipole microstrip line discontinuities (meandered line), the current magnitude has the tendency to increase at the edge of the meandered lines. Nevertheless, the square loop surface has very low current flowing over the structure. Fig 12 (B) demonstrates that for a 90° phase change of the current source, the current flow’s direction reverts. The feed-line characteristics are found to be moderately similar for 0°. The meandered lines at both sides of the antenna, however, do not appear to respond well to the 90° phase difference of the current flow. The square loop connecting both the meandered lines possesses a current flow over its surface when the current flow is 90° out of phase. The current over the meandered lines is flowing, as the path is revealed by the vector lines. Given this type of current distribution, the antenna has a circular radiation pattern that is normal to the antenna.

The tapered feed-line, with the aid of the square loop, works as the impedance matching network conjugate to the ASIC. Here, the tapered line is defined by two characteristics: length and the tapered function. Optimization of these two characteristics leads towards the current dimensions of the tapered feed-line for the proposed antenna. The current density change within the tapered feed-line depicts the change of current distribution within the dimensions of the antenna.

**Fig 13:**
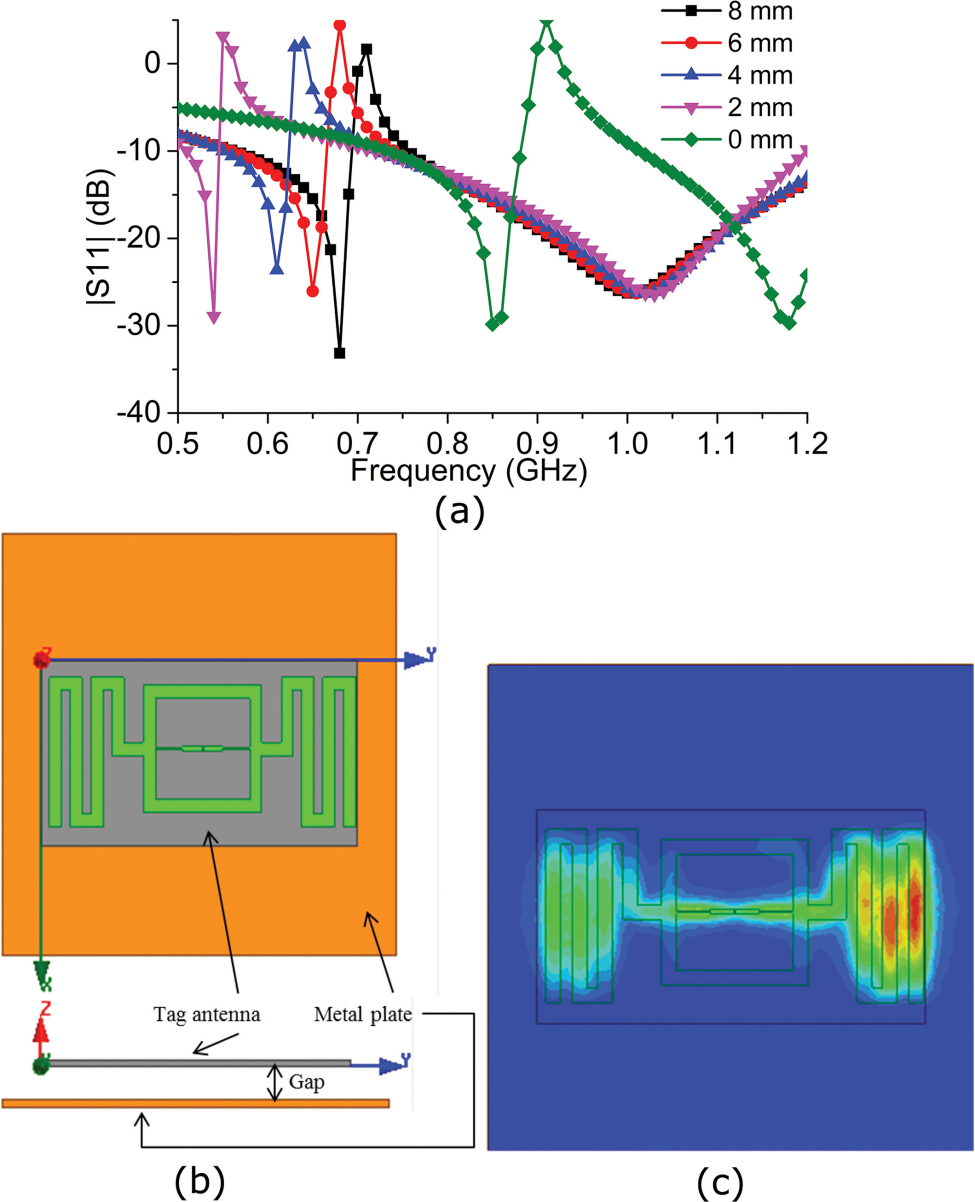
Effect on |S_11_| after change of distance from the metal plate towards the tag.

A metal plate with a thickness of 2 mm and an area of 100 mm×100 mm^2^ is attached to the back of the tag antenna to discover the antenna’s metallic near field behavior. Iron is selected as the metal plate, with a relative permittivity of 1 and a relative permeability of 4000 (ferromagnetic). Iron has a bulk conductivity of 10300000 Siemens/m and a mass density of 7870. Fig 13 (A) demonstrates the tag antenna’s reflection coefficient due to the change in the distance between the metal layer and the tag antenna. The figure reveals that the reflection coefficient remains almost unchanged as the distance is reduced from 8 mm to 4 mm. The antenna impedance has the tendency to change for a metal distance of 2 mm; however, within the UHF RFID band, the impedance change is hardly noticeable. Similarly, when the distance is minimized to 0 mm, a notch is introduced at the middle of the UHF RFID frequency band. A frequency ranging from 840 MHz to 880 MHz is much lower than the -10 dB reflection coefficient. When the tag antenna is attached to the metal layer (distance of 0 mm), it begins to attract a magnetic or electromagnetic field towards it because of the high permeability of iron. The iron’s permeability is the measure of its magnetization; it becomes effective when it is inside the near field of any electromagnetic or magnetic field, thus retrieving the magnetic energy from the antenna and having an effect on its resonance frequency. Fig 13 (B) demonstrates the setup of the metallic plate based on the tag antenna. Fig 13 (C) demonstrates the antenna’s current distribution pattern when the metallic plate is at a distance of 0 mm from the tag antenna. It is observed that most of the current flow is attracted into the metallic plate from the meandered lines of the tag antenna.

**Fig 14:**
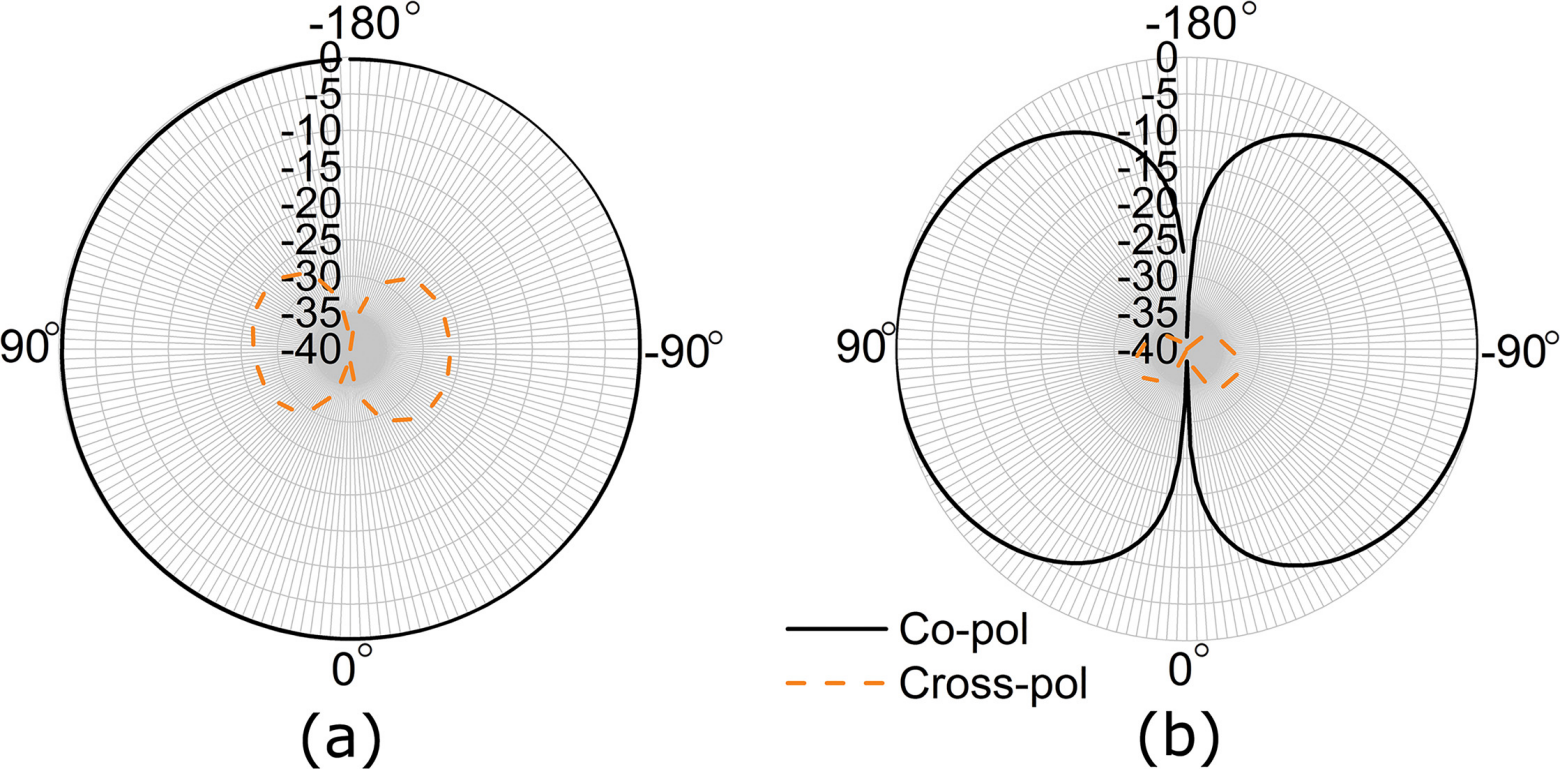
Normalized (a) E-plane and (b) H-plane radiation pattern of the tag antenna.

Fig 14 demonstrates the tag antenna’s radiation pattern. It can be observed from the normalized E-plane co-pol radiation pattern in Fig 14 (A) that the radiation pattern follows an omnidirectional pattern. Nevertheless, the cross-pol radiation pattern that is normalized at the E-plane of the tag antenna reveals two introvert points at -180° and 0°. Fig 14 (B) demonstrates the antenna’s H-plane radiation pattern. Both the co- and cross-pol radiations at the H-plane of the tag antenna reveal almost the same pattern following the normalization process. Based on the antenna’s 3D radiation pattern at the H-plane, the proposed tag antenna produces a donut-shaped radiation pattern. It is found that both the co- and cross-pol radiation patterns at the H-plane have nulls at -180° and 0°, producing a donut-shaped radiation pattern for the tag antenna.

A tag antenna’s essential realization and evaluation is the distance of the read range from the reader antenna. The antenna’s read range can be measured from the equation as shown in the following [16]:

Read_range=DPePtagGRLcable (3)

where *D* = the distance between the tag antenna and the reader antenna in the measurement system, *P*_*e*_ = the effective isotropic radiated power (EIRP) (differing in terms of region), *G*_*r*_ = the realized gain of the reader antenna, *L*_*cable*_ = the power loss caused by the connection cable, and *P*_*tag*_ = the minimum power needed to excite the antenna’s tag.

**Fig 15:**
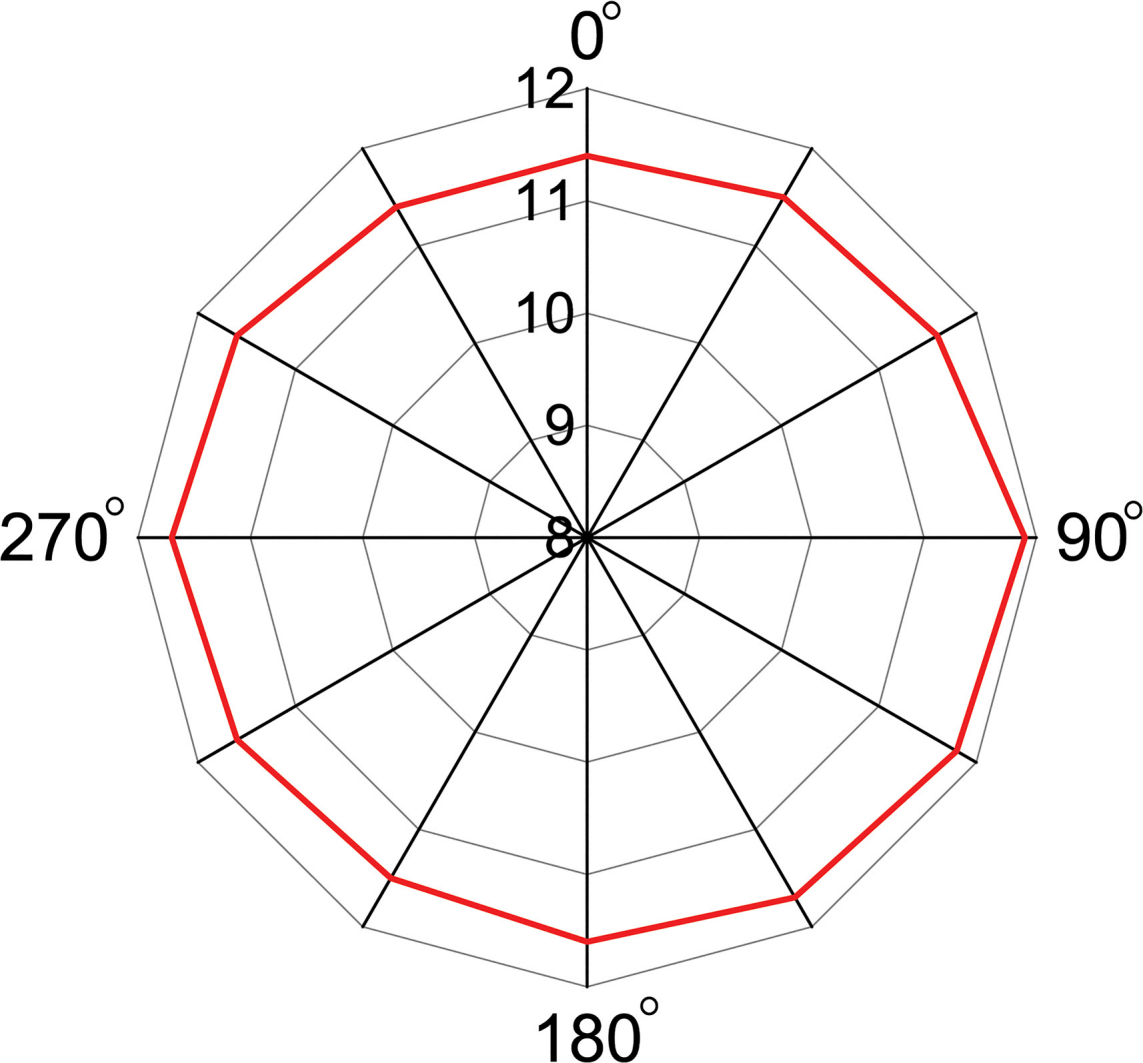
Read range of the antenna at zx-plane.

The desired read range characteristics depend entirely on the RFID applications. Nevertheless, long-range tag detection is preferred in most of the applications. The NXP ASIC used for this passive tag antenna design contains a maximum sensitivity of -18 dBm. A reader module of model no. R420 by Impinj is connected to a horn antenna with model no. SAS-571. The horn antenna’s performance is calibrated for a distance of 10 meters. The horn antenna has a gain of 7.6 dBi at 900 MHz. The Malaysian Communications and Multimedia Commission provides the guideline that a maximum EIRP power of 3.2 W can be used as the transmission power for the reader antenna. Fig 15 demonstrates the antenna’s measured read range using a 3.2-W EIRP power at the zx-plane. The read range is taken towards the normal of the antenna. A maximum read range of 11.9 meters is reached from the proposed tag antenna with stable performance.

**Fig 16:**
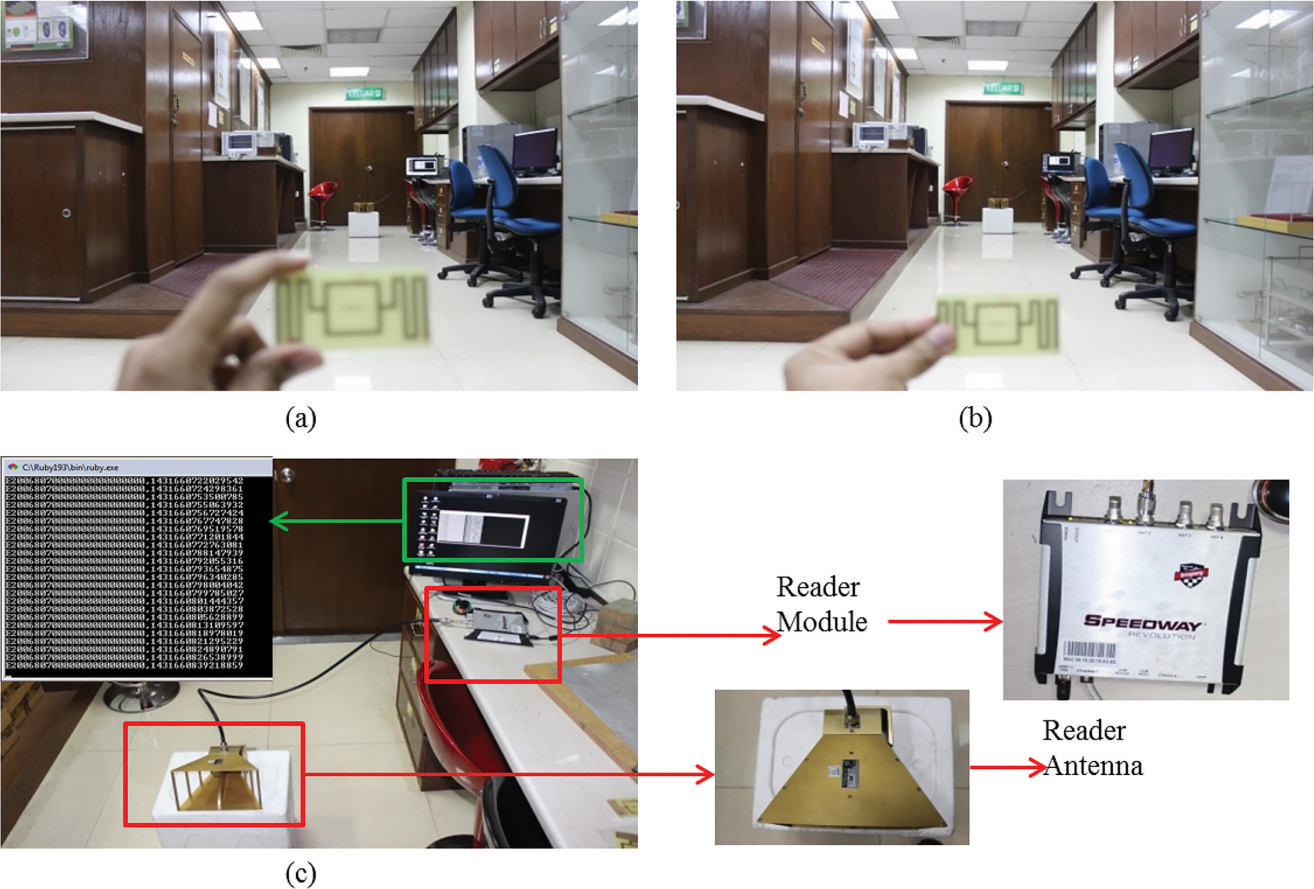
Read range measurement setup (a) without touching the radiating plane, (b) touching the radiating plane, (c) tag chip id reading.

If the antenna is held outside the radiating patch, as shown in Fig 16 (A), the antenna response is the same as in Fig 15. If the antenna is held by touching the radiating plane, as shown in Fig 16 (B), the read range of the antenna tends to increase, changing the characteristics of the antenna. The human body, when connected with the antenna’s radiating element, works as an extension of the radiating element and helps absorb the power. There are also many other reasons for the read range to change [15]. To overcome this characteristic change, the antenna can be shielded using proper radome, which will aid the antenna in avoiding direct connection with the human body [17, 18].

Table 1 depicts the comparison between the literature and the proposed antenna. It can be observed from the table that the antennas in the literature takes more space on average, compared to the proposed antenna. Moreover, the read range is as high as 11.9 m for the proposed antenna, which is higher compared to the other antennas in Table 1.

**Table 1:** Tag antenna specifications from the literatures.

Antenna	Substrate	Dimension (mm^3^)	Free space Read Range (m)	Mountable on Metallic surface
[2]	Rogers RB4350B (Ԑ_r_ = 3.66)	63 × 47 × 1.524	6.38	No
[3]	FR4 (Ԑ_r_ = 4.4)	96 × 50 × 2	4	Yes
[5]	FR4 (Ԑ_r_ = 4.6)	76.5 × 22 × 3	6.5	Yes
[6]	PET (Ԑ_r_ = 3.4)	91 × 27 × 0.07	7.5	Yes
[11]	FR4 (Ԑ_r_ = 4.4)	70 × 70 × 1.63	3	Yes
**Proposed Antenna**	**FR4 (Ԑ**_**r**_ **= 4.6)**	**80 × 44 × 1.6**	**11.9**	**Yes**

## Conclusion

A microstrip co-planar type of RFID tag antenna with passive and simple characteristics covering the UHF RFID frequency is demonstrated in this study. The antenna has a low profile with a structure of meandered lines at both sides of the tag. The tag antenna is designed to match the conjugate chip impedance of the NXP SL3S1213 UCODE G2iL UHF RFID chip with an impedance of 23-j224 Ω. An omnidirectional radiation pattern that is donut-shaped is achieved with a maximum realized gain of 1.43 dBi at 860 MHz. A balun of λ/4 length is used to calculate the antenna’s reflection characteristics. Current distribution patterns are demonstrated to assess the field effects at various phases of the current flow. A maximum read range of 11.9 meters is yielded with this tag antenna. An analysis is given based on the near field metallic layer effect on the tag antenna. The tag antenna is designed using an inexpensive one-sided FR4 substrate. The proposed tag antenna is efficient, cost-effective, and compact, thus proving to be a considerable candidate for the UHF passive RFID tag system.
